# Recent Advances in Non-invasive Brain Stimulation for Major Depressive Disorder

**DOI:** 10.3389/fnhum.2017.00526

**Published:** 2017-11-06

**Authors:** Shui Liu, Jiyao Sheng, Bingjin Li, Xuewen Zhang

**Affiliations:** Jilin Provincial Key Laboratory on Molecular and Chemical Genetics, The Second Hospital of Jilin University, Changchun, China

**Keywords:** non-invasive brain stimulation, repetitive transcranial magnetic stimulation, transcranial direct current stimulation, affective processing, major depressive disorder

## Abstract

Non-invasive brain stimulation (NBS) is a promising treatment for major depressive disorder (MDD), which is an affective processing disorder involving abnormal emotional processing. Many studies have shown that repetitive transcranial magnetic stimulation (rTMS) and transcranial direct current stimulation (tDCS) over the prefrontal cortex can play a regulatory role in affective processing. Although the clinical efficacy of NBS in MDD has been demonstrated clinically, the precise mechanism of action remains unclear. Therefore, this review article summarizes the current status of NBS methods, including rTMS and tDCS, in the treatment of MDD. The article explores possible correlations between depressive symptoms and affective processing, highlighting the relevant affective processing mechanisms. Our review provides a reference for the safety and efficacy of NBS methods in the clinical treatment of MDD.

## Introduction

Major depressive disorder (MDD) is one of the most common and disabling mental disorders, and has become the second leading contributor to the global disease burden (Collins et al., 2011; Whiteford et al., 2013; Otte et al., 2016). MDD is characterized by maladaptive and persistent emotional responses to stressors (Groenewold et al., 2013). Because of its high incidence and common recurrence, MDD represents a serious challenge for world public health. Currently, approximately 1/3 of MDD patients globally exhibit treatment-resistant depression, because of invalid or ineffective antidepressant treatment (Rush et al., 2006).

Affective processing is crucial for the basic tasks of human survival and adaptation, involving many functions including perception, attention, learning, memory and responses to the environment (Narumoto et al., 2001; Garrett and Maddock, 2006; Del Piero et al., 2016). Dysfunctional affective processing is considered a key factor in the occurrence and development of many psychiatric disorders, including anxiety, schizophrenia and bipolar disorder (Anderson et al., 2017; Bocharov et al., 2017; Krakowski and Czobor, 2017; Wolkenstein et al., 2017). Previous studies have suggested that MDD is associated with dysfunctional processing in affective-related neural circuits (Clark et al., 2009). Meanwhile, cognitive abnormalities are also a core feature of depression, which involves many domains including attention, memory, executive functions and psychomotor speed (Gonda et al., 2015). Beck proposed a cognitive model of depression in which negative stimuli in the environment can attract conscious or unconscious attention, and patients with depression tend to make negative evaluations of themselves and others, suggesting that depression might be caused by negative cognitive schemas (Disner et al., 2011). For example, hopelessness, manifested as overestimating the damage of a negative event and underestimating the positive outcome of the future, is thought to be an important cognitive risk factors of depression (Wang et al., 2015). Many behavioral studies have demonstrated that patients with MDD exhibit a negative emotional bias, manifesting as preferential processing of negative over positive stimuli, in accord with Beck’s hypothesis (Erickson et al., 2005; Leyman et al., 2007; Yang et al., 2011). Strunk and Adler (2009) examined the relationship between depressive symptoms and bias, reporting that patients high in depressive symptoms exhibited significant pessimistic bias on three judgment tasks. The modern cognitive neuropsychological model of depression is a reformulation and expansion of Beck’s cognitive model of depression, and the results are derived from pharmacological studies and concerning basic neurocognitive function. This model also proposes that patients with depression may develop an alteration in the bottom-up emotional stimulus processing, leading to negative perception (Roiser et al., 2012; Gonda et al., 2015).

Recent evidence suggests that negative affective and cognitive processing bias of MDD patients may originate from structural and functional abnormalities in specific brain regions, including the dorsolateral and ventral prefrontal cortex, hippocampus and amygdala, which are associated with affective processing (Campbell et al., 2004; Hamilton et al., 2008; Koenigs and Grafman, 2009). Numerous studies have demonstrated that the networks abnormality is one crucial mechanism in the occurrence and development of MDD, which underlies altered affective and cognitive processing, such as increased reactivity as well as increased attentional and cognitive bias towards negative stimuli in MDD (Hamilton et al., 2012; Groenewold et al., 2013). And the default mode network (DMN), the executive control network (ECN), and the salience network (SN) are three major networks in the recent studies of MDD. The DMN is involves in self-referential processing and episodic memory retrieval (Raichle et al., 2001). The ECN is involved in executive function and emotion regulation, with functional regions being the dorsolateral prefrontal cortex (DLPFC) and lateral posterior parietal regions (Miller and Cohen, 2001). The core regions in the SN include amygdala, and anterior hippocampus, which is involved in detection of, and direction of attention to, salient environmental stimuli (Chen and Etkin, 2013). Some studies revealed functional connectivity changes in the ECN and SN in depressed patients (Bonavita et al., 2017), which suggested the potential links between networks abnormality and depressive symptoms. Studies found that the enhancement of affective processing in MDD manifested excessive activation of the amygdala and supragenual cingulate of the brain (Matthews et al., 2008; Davey et al., 2011). In addition, the deficiency of cognitive control in MDD has demonstrated the correlations with insufficient activity in the DLPFC and anterior cingulate gyrus (Holmes and Pizzagalli, 2008; McNeely et al., 2008). These findings suggest that during the affective processing of depressive individuals, networks abnormalities, manifested as enhanced affective processing and decreased cognitive control function, might result in a more intense experience of negative emotion, inducing depression.

Non-invasive brain stimulation (NBS) methods have been found to be effective for regulating human brain function, including transcranial magnetic stimulation (TMS), transcranial direct current stimulation (tDCS) and transcranial alternating current stimulation (tACS). In the past few decades, NBS techniques have been found to be useful for regulating healthy individual control of consciousness, and positive therapeutic effects have been reported for a range of psychiatric disorders, including depression, Alzheimer’s disease, and epilepsy (Kuo et al., 2014; De Raedt et al., 2015). As NBS techniques develop, they have been applied in the clinical treatment of MDD, and the regulatory effects of NBS on affective processing have been consistently verified (Nitsche et al., 2012; Conson et al., 2015).

In the current review, we discuss the possible mechanisms by which NBS methods, including repetitive TMS (rTMS) and tDCS, improve depressive symptoms by modulating affective processing. Moreover, we examine research investigating the value of combining NBS with imaging techniques to improve antidepressant effects. Thus, this review article can provide a reference for the safety and efficacy of NBS methods in the clinical treatment of MDD.

## Repetitive Transcranial Magnetic Stimulation (rTMS)

### rTMS Overview

TMS is an NBS technique that was first created in 1985 by Brunoni et al. (Valero-Cabré et al., 2017). In TMS, electromagnetic induction is used to focus a current and modulate cortical function (Hallett, 2007). TMS devices consist of a capacitor to store charge and a stimulation coil to transfer energy. When the charge capacitor is rapidly released, the generated current passes through the stimulating coil to produce magnetic lines of flux with low resistance and no trauma, penetrating the skull to reach the cortex and reverse the current conduction in the cortex, thereby altering cortical excitability (Noda et al., 2015). rTMS is a new neurophysiological technique based on TMS, involving the delivery of repetitive stimuli at a specific cortical site. Previous studies have shown that low-frequency stimulation of rTMS (≤1 Hz) can reduce the excitability of neurons and inhibit cortical activity, whereas high-frequency stimulation (≥5 Hz) can increase the excitability of neurons and enhance cortical activity (Mitchell and Loo, 2006; Milev et al., 2016). To date, rTMS has been approved as a clinical therapy for MDD in several regions, including the USA, Canada the European Union (Tortella et al., 2014).

### Affective Processing-Related Mechanisms of rTMS in Antidepressant Treatment

Previous studies have indicated that multiple brain regions, including the amygdala, prefrontal cortex, parietal lobe and modality-specific sensory cortex regions, play distinct roles in regulating emotional processing (Dalgleish, 2004; Pessoa, 2017). The valence hypothesis proposes that affective processing exhibits hemispheric lateralization, with the right hemisphere specializing in negative emotion processing and the left hemisphere specializing in positive emotion processing (Prete et al., 2015). This hypothesis has been supported by neuroimaging studies (Grimm et al., 2008), and several previous studies have shown that the DLPFC influences emotional stimulus categorization, emotional evaluation, emotional memory, and emotional regulation (Brennan et al., 2017; Zilverstand et al., 2017). Thus, the DLPFC is thought to play a leading role in emotional control. Previous studies reported that activation of the left DLPFC is associated with processing positive emotions, whereas activation of the right DLPFC is thought to be responsible for processing negative emotions (Mondino et al., 2015). Schutter and van Honk (2005) found that, in patients with depression, left DLPFC responses were decreased and right DLPFC responses were increased.

rTMS is a relatively localized intervention, and several studies have examined its potential role in antidepressant treatment, with the DLPFC as a primary target (Lepping et al., 2014; Serafini et al., 2015; Carle et al., 2017; Carpenter et al., 2017). Two rTMS protocols are commonly used for treating MDD: high-frequency rTMS (10–20 Hz) targeting left DLPFC, and low-frequency rTMS (≤1 Hz) targeting right DLPFC (Isenberg et al., 2005). Zwanzger et al. (2014) reported that inhibitory rTMS over the right DLPFC could improve and regulate affective processing, indicating that rTMS might exert an antidepressant role via affective processing-related mechanisms. A study of the role of frontal stimulation in emotional processing by Vanderhasselt et al. (2009) revealed that high-frequency rTMS applied to left DLPFC can improve task-switching abilities in depressed individuals. Moreover, clinical evidence has indicated that low-frequency rTMS over right DLPFC can increase response rates to monotherapy for MDD (Berlim et al., 2013b). These findings are consistent with the valence hypothesis, providing strong support for the notion that the antidepressant effects of rTMS involve the regulation of affective processing.

At the same time, some other brain regions associated with affective processing, including dorsomedial prefrontal cortex (DMPFC), frontopolar cortex (FPC), ventromedial prefrontal cortex (VMPFC), and ventrolateral prefrontal cortex (VLPFC), have been also considered as potential targets for clinical application of rTMS (Downar and Daskalakis, 2013; Junghofer et al., 2017). Of these, DMPFC received the most attention to date. Bakker et al. (2015) found that DMPFC-rTMS could show a similar antidepressant effect of DLPFC-rTMS in patients with MDD. Case series in MDD and bipolar disorder have provided initial evidence that DMPFC-rTMS may be safe, tolerable and effective in antidepressant treatment (Downar and Daskalakis, 2013; Downar et al., 2014). Nevertheless, there is a need for more researches and clinical trials of DMPFC as a target for clinical application of rTMS in MDD.

### Combination of rTMS and Antidepressants in the Treatment of MDD

Previous studies have indicated that rTMS can lead to long-term and sustained remission of treatment-resistant MDD, significantly improving the quality of life and functional status of MDD patients (Galletly et al., 2016; Teng et al., 2017). Moreover, some studies have found that rTMS may improve antidepressant effect in combination with traditional antidepressants (Table 1). In a study by Wang et al. (2017), 43 patients with first-episode MDD were randomly divided into two groups. Subsequently, active or sham rTMS was applied to the left DLPFC, and a 4-week course of combination therapy with paroxetine was administered. The results indicated that patients in the active rTMS group had a higher response rate than those in the sham rTMS group at the end of the fourth week, and the remission rate in the experiment group was clearly elevated compared with the control group (Wang et al., 2017). These results suggest that rTMS might enhance the response of depressed patients to paroxetine, enhancing antidepressant efficacy. A double-blind clinical randomized controlled trial (RCT) by Huang et al. (2012) also confirmed the efficacy of rTMS in combination with conventional antidepressants in the treatment of depression. In their study, 60 patients with first-episode MDD were randomly categorized into two groups. In the first 2 weeks, patients in the two groups were treated with active or sham rTMS combined with escitalopram treatment, followed by another 2 weeks of escitalopram monotherapy. The results revealed that, compared with the control group, scores on the 17-item Hamilton Depression Rating Scale (HAMD-17) dropped more than 20% in the active rTMS group in the first 2 weeks. Furthermore, the active rTMS group exhibited a significantly faster score reduction compared with the sham group at 2 weeks, suggesting that rTMS had a synergistic effect in the treatment of MDD with traditional antidepressants.

**Table 1:** Comparison between non-invasive brain stimulation (NBS) combined with antidepressants and individually NBS or antidepressants for major depressive disorder (MDD) in randomized controlled trials (RCTs).

Experimental group	Control group	Sample size (N)	Subjects	Sessions duration and frequency (total sessions)	Stimulation site	Assessment	Main results	Reference
Active rTMS combined with paroxetine	Sham rTMS combined with paroxetine	43	Patients with first major depressive disorder	10 Hz, 20 sessions for 4 weeks	left DLPFC	HAMD-24	A significant improvement in the HAMD-24 after active rTMS combined with paroxetine vs. sham rTMS combined with paroxetine	Wang et al. (2017)
Active rTMS combined with escitalopram	Sham rTMS combined with escitalopram	60	Patients with first major depressive disorder	10 Hz, 10 sessions for 2 weeks	DLPFC	HAMD-17, MADRS	A significant improvement in the HAMD-17 after active rTMS combined with escitalopram vs. other control groups	Huang et al. (2012)
Active tDCS combined with sertraline	Sham tDCS combined with sertraline; active tDCS combined with placebo; sham tDCS combined with placebo	120	Patients with major depressive disorder	2 mA, 12 sessions for 6 weeks	DLPFC	HAMD-7, MADRS, BDI and CGI.	A significant improvement in the HAMD-7, MADRS, BDI and CGI after active rTMS combined with sertraline vs. other control groups	Brunoni et al. (2013)
Active rTMS combined with escitalopram	Sham-controlled rTMS combined with escitalopram	45	Patients with medica- tion-resistant major depression	8 Hz, 15 sessions for 3 weeks	Left DLPFC	HAMD-6, HAMD-17 and MES	A significant improvement in the HAMD-6 after active rTMS combined with escitalopram vs. the control group	Bretlau et al. (2008)
Active rTMS combination with amitriptyline	Sham-controlled rTMS combined with amitriptyline	46	Patients with non-psychotic depressive episode	5 Hz, 20 sessions for 4 weeks	Left DLPFC	HAMD-17, MADRS, VAS and CGI	A significant improvement in the HAMD-17, MADRS, VAS and CGI after active rTMS combined with amitriptyline vs. the control group	Rumi et al. (2005)
Active rTMS combination with venlafaxine, sertraline or escitalopram	Sham rTMS combination with venlafaxine, sertraline or escitalopram	99	Patients with major depressive disorder	15 Hz, 10 sessions for 2 weeks	Left DLPFC	HAMD	A significant improvement in the HAMD after active rTMS combined with antidepressants vs. other control groups	Rossini et al. (2005)

*Abbreviation: HAM-D, Hamilton Depression Rating Scale; MES, Bech-Rafaelsen Melancholia scale; VAS, Visual Analog Scale; MARDS, Montgomery-Asberg depression rating scale; BDI, Beck Depression Inventory; CGI, Clinical Global Impression*.

### Comparison of rTMS and ECT in Antidepressant Treatment

Electroconvulsive therapy (ECT) has been used for the treatment of human diseases for more than 80 years, and is currently considered the most effective treatment for MDD (UK ECT Review Group, 2003). At present, the main technique used in clinical settings is modified ECT (MECT). This method involves the administration of anesthetics and muscle relaxants before treatment, so that the electrical stimulation does not cause convulsions, which in turn results in the elimination of muscle rigidity and tremor, as well as avoiding fracture, dislocation and other complications (Liu et al., 2016). Although ECT has been shown to be effective in the short term, its recurrence rate, particularly the high rate of early recurrence, and the cognitive side effects are important challenges in this form of antidepressant treatment (Jelovac et al., 2013; Fernie et al., 2014). A meta-analysis reported that despite continued drug treatment after ECT treatment, the relapse rates was 51.1% in the first year after treatment, peaking in the first 6 months, up to 37.7% (Jelovac et al., 2013).

There are clear differences in antidepressant mechanism, tolerance and acceptability between rTMS and ECT (Table 2). The antidepressant effects of rTMS and ECT in MDD have been compared in numerous studies (Möbius et al., 2017). Chen et al. (2017) conducted a meta-analysis including 25 clinical RCTs involving 1288 MDD patients. The findings revealed that the therapeutic effects of ECT were greater than those of rTMS, but right prefrontal-rTMS had the best tolerance. Jin et al. (2016) performed a retrospective study of 150 MDD patients receiving MECT and 150 MDD patients receiving rTMS, showing that in the short-term, the response rate in the MECT group was higher than that in the rTMS group, although there was no clear difference in long-term relapse-free survival between groups. Furthermore, the cost benefit of ECT was found to be higher than that of rTMS, and, because of its non-invasive and convenient features, as well as its minimal side effects relative to ECT, rTMS was favored by patients (Magnezi et al., 2016). Nevertheless, as an emerging treatment technology for antidepressant therapy, further in-depth clinical studies of rTMS are required before it becomes a widespread alternative to ECT.

**Table 2:** Comparison among rTMS, tDCS, ECT in the treatment of MDD.

	Repetitive transcranial magnetic stimulation (rTMS)	Transcranial direct current stimulation (tDCS)	Modified electroconvulsive therapy (MECT)
Contraindications	Cochlear implants, brain stimulators or electrodes, aneurysm clips; Implantable electronic devices (such as pacemakers, etc.), implantable defibrillator, a history of epilepsy, or the presence of a brain lesion (vascular, traumatic, neoplastic, infectious, or metabolic)	Implantable electronic devices (such as pacemakers, etc.), serious heart disease, acute large area of cerebral infarction, irritation area with hyperalgesia, increased intracranial pressure, pregnant women, vital signs instability, bleeding tendency patients	Implantable electronic devices (such as pacemakers, etc.), intracranial infections, intracranial tumors, intracranial metal, head trauma, serious heart disease, acute large area of cerebral infarction, irritation area with hyperalgesia, increased intracranial pressure, pregnant women, infants, vital signs instability, bleeding tendency of patients
Mechanism of action	The LTP-like and LTD-like effects of rTMS rely on NMDA receptor–mediated glutamatergic function	Modifying synaptic strength NMDA receptor-dependently or altering GABAergic activity (reduced)	Seizure induced changes in neurotransmitters, neuroplasticity and functional connectivity
Stimulation site and delivery parameters	High-frequency stimulation (10–20 Hz) over left DLPFC; low-frequency stimulation (≤1 Hz) over right DLPFC	Anodal stimulation over left DLPFC; cathodal stimulation over right DLPFC or right OFC (1–2 mA)	Bilateral treatments (both bitemporal and bifrontal) most often use 1.5–2.0 times seizure threshold (ST) and right unilateral 5–6 or even eight times ST
Side effects	Scalp pain during stimulation, transient headache, seizure induction, transient hearing loss	Redden of the skin, itching, burning, heat and tingling sensations at the stimulation site	Headache, muscle soreness, nausea and myalgia, cognitive impairment (such as retrograde amnesia)
Advantages	Non-invasive, lower cost	Non-invasive, portable, easy to use, long-acting, lowest cost	Most effective among three therapies

### Disadvantages and Side Effects

The most common side effects of rTMS in clinical settings include headache (5%–23%) and discomfort at the stimulus site (20%–40%), and the most severe side effect is the induction of seizures (Machii et al., 2006; Maizey et al., 2013; Wall et al., 2014; Dobek et al., 2015; Boes et al., 2016). Prikryl and Kucerova (2005) reported a case of generalized tonic clonic seizure in a patient with MDD receiving rTMS. To date, fewer than 25 cases of rTMS-induced seizure have been reported worldwide. Therefore, high frequency rTMS is contraindicated in patients with a history of seizures, although the incidence rate is relatively low (<0.1%; Dobek et al., 2015).

## Transcranial Direct Current Stimulation (tDCS)

### tDCS Overview

tDCS is an NBS method acting on specific cortical areas by producing a persistent, weak, direct current (usually 1–2 mA) through electrodes placed on the skull (Blumberger et al., 2015). The basic principle is that stimuli with different polarities can cause changes in the hyperpolarization or depolarization of the resting membrane potential. Anodic stimulation can improve the excitability of the cortex through the depolarization of the membrane potential, while cathodic stimulation can help reduce cortical excitability via hyperpolarization of neuronal membrane potentials (Stagg and Nitsche, 2011). Previous studies have indicated that the neurophysiological mechanisms of tDCS may involve subliminal regulation of the resting membrane potential of neurons inducing a polarity-dependent modification of N-methyl-d-aspartate (NMDA) receptor function (Nitsche et al., 2003). Because NMDA receptor function is involved in synaptic plasticity formation, this can result in the production of neural remodeling and changes in the excitability of the cortex during stimulation (Nitsche et al., 2003). The stimulation of tDCS is weak, but it can also cause changes in cortical excitability, and the effect lasts longer after stimulation than that of rTMS. A previous study reported that following current stimulation of the body for several minutes, changes in cortical excitability can last for approximately 1 h after stimulation (Nitsche and Paulus, 2000). Meanwhile, compared with rTMS, tDCS has the advantages of portability, low equipment cost and minimal adverse reactions (Lefaucheur et al., 2017; Table 2).

### Affective Processing-Related Mechanisms of tDCS in Antidepressant Treatment

The DLPFC is one of the major brain areas involved in emotion regulation (Baeken et al., 2010), and various neuroimaging studies have indicated that it plays an important role in top-down regulation of affective processing (Disner et al., 2011). Some studies have suggested that DLPFC activity can be mediated by tDCS, thus playing a regulatory role in affective processing (Boggio et al., 2009; Sanchez et al., 2016). Single-session tDCS studies in healthy samples by Utz et al. (2010) revealed acute improvement in affective and cognitive processing. Further research has confirmed that the DLPFC plays an important role in the occurrence and development of depression (Schutter and van Honk, 2005). Imaging studies have also shown that left DLPFC cerebral blood flow and metabolism are decreased in patients with depression, while the right DLPFC exhibits increased metabolic activity (Shiozawa et al., 2015).

A number of studies have confirmed the role of tDCS in antidepressant treatment (Vigod et al., 2014; Al-Kaysi et al., 2017; Brennan et al., 2017). At present, the left and right DLPFC are typically used as anode and cathode stimulation sites for the majority of tDCS treatment methods, which can increase the excitability of the left DLPFC and inhibit the excitability of the right DLPFC to alleviate depressive symptoms (Meron et al., 2015). Through a double-blind RCT, Wolkenstein and Plewnia (2013) detected the effect of a single-session anodal tDCS targeting the left DLPFC in MDD patients, reporting a significant improvement in emotional cognitive control. This finding provided further evidence that tDCS might improve depressive symptoms by modulating emotional processing. Brunoni et al. (2011, 2013, 2014) conducted a double-blind RCT involving 24 depressive patients, and presented the emotional Stroop task, measuring response times (RTs) to positive-, negative-, and neutral-related words. The results revealed that active tDCS significantly modified negative attentional bias, abolishing the RT delay for negative words (Brunoni et al., 2014). This finding suggests that the regulatory effect of tDCS on affective processing might be an important mechanism underlying the antidepressant effects of the treatment method.

### Combination of tDCS and Antidepressants in the Treatment of MDD

Some studies have found that tDCS combined with traditional antidepressants might have a synergistic therapeutic effect (Table 1). Brunoni et al. (2011, 2013, 2014) conducted a double-blind RCT, dividing participants into four groups using pairwise combinations of sertraline/placebo and active/sham tDCS. When Montgomery-Asberg Depression Rating Scale (MADRS) scores were measured, the results revealed that combined treatment was significantly superior to placebo, tDCS only, and sertraline only. There was no significant difference in side effects between different modalities of intervention (Brunoni et al., 2013), indicating that the combination of tDCS and antidepressants in patients with MDD performed better than applying either treatment alone. These findings may provide a new direction for the widespread application of tDCS in MDD treatment.

### Disadvantages and Side Effects

According to the current safety guidelines of tDCS, the adverse effects are minimal for both healthy individuals and MDD patients, regardless of whether tDCS is applied to the motor areas or non-motor areas of the cortex. Reddening of the skin, heat, burning, itching, and tingling sensations at the stimulation site are the most common side effects of the treatment, and are reported by more than half of patients receiving tDCS (Brunoni et al., 2011; Shiozawa et al., 2014; Meron et al., 2015). Brunoni et al.’s (2011) systematic review of 117 studies conducted between 1998 and 2010 investigated the adverse effects of tDCS on the human brain, reporting that slight itching and tingling were the main adverse events, and that retention time was transient.

### Prospects for NBS Techniques in Antidepressant Treatment

The efficacy of NBS in treatment remains limited, even though its effectiveness in improving depressive symptoms in MDD patients has been consistently validated (McLoughlin et al., 2007; Berlim et al., 2013a; Lefaucheur et al., 2017). As a local brain stimulation technique, the therapeutic efficacy of NBS depends largely on the choice of stimulation sites and the accuracy of the location (Herbsman et al., 2009; Fox et al., 2012). Several previous studies have indicated that imaging-guided NBS could help to locate specific functional brain networks at a higher resolution. Using this approach, stimulation sites can be individually and accurately positioned according to anatomical differences of individual depressed patients, thereby improving the therapeutic effectiveness of NBS in antidepressant treatment, and supporting the extensive application of NBS approaches in the clinic (Mir-Moghtadaei et al., 2015; Luber et al., 2017).

Jha et al. (2016) examined the effects of a 4-week treatment regime in refractory MDD patients with single-photon emission computed tomography (SPECT) guided high-frequency rTMS and standard high-frequency rTMS. In their experiment, subjects were assessed with the MADRS, the Beck Depression Inventory (BDI) and the Clinical Global Impression (CGI) scale. The response rate of the subjects in the brain SPECT guided group was found to be significantly higher than that in the standard group, on MADRS, BDI and CGI scores (Jha et al., 2016). These findings indicate that rTMS combined with brain SPECT targeting specific brain regions could improve antidepressant treatment in clinical settings.

An important topic for the future development of NBS is determining the combinations of imaging methods that provide optimal antidepressant treatment effects, to develop individualized treatment for MDD patients.

## Limitations

Several limits of this systematic review should be acknowledged. First, a common limitation to the research presented in this review is the widespread differences in measurement tools used to measure depressive symptoms and identify depression. A large number of different scales for measuring depressive symptoms were used across different studies, including the HAM-D, MES, VAS, MARDS, BDI and CGI, which may produce different amounts of measurement error in different samples depending on the population in which they are being used. Second, sample size of some RCTs in the review is relatively small. Finally, this review may be limited by reporting bias, the under-reporting of undesirable or non-significant experimental results. This may have leaded to lacking negative reports on the association between NBS and MDD, further weakening the evidence against a role of NBS in MDD.

## Conclusion

As an emerging non-invasive antidepressant treatment approach with few adverse reactions, NBS techniques have been extensively studied since their inception, and their clinical application in the treatment of MDD is increasing. Studies have indicated that the development of MDD may be closely related to abnormal affective processing (Harmer et al., 2009). Brunoni et al. (2014) found that one single active bi-frontal tDCS significantly modifies negative attentional bias in MDD. Other studies found that NBS including tDCS and rTMS can improved deficient cognitive control, further enhancing affective processing in MDD (Hoy et al., 2012; Wolkenstein and Plewnia, 2013). In a word, NBS may alleviate the symptoms of depression by regulating affective processing and enhancing cognitive control. As research progresses, it is likely that the antidepressant mechanisms of NBS will become more specific, the corresponding treatment effects will continue to improve, and its applications in MDD treatment will become more extensive.

## Author Contributions

SL and JS wrote the manuscript. XZ and BL provided the critical revisions. All authors approved the final version of the manuscript for submission.

## Conflict of Interest Statement

The authors declare that the research was conducted in the absence of any commercial or financial relationships that could be construed as a potential conflict of interest.
